# Learning in a Virtual World: Experience With Using Second Life for Medical Education

**DOI:** 10.2196/jmir.1337

**Published:** 2010-01-23

**Authors:** John Wiecha, Robin Heyden, Elliot Sternthal, Mario Merialdi

**Affiliations:** ^4^Department of Reproductive Health and ResearchWorld Health OrganizationGenevaSwitzerland; ^3^Section of Endocrinology, Diabetes, and NutritionDepartment of MedicineBoston Medical CenterBoston MAUSA; ^2^Education ConsultantWellesleyMAUSA; ^1^Department of Family MedicineBoston University School of MedicineBoston Medical CenterBostonMAUSA

**Keywords:** Medical education, continuing medical education, computer-assisted instruction, computer aided instruction, distance education, computer simulation, patient simulation, diabetes mellitus

## Abstract

**Background:**

Virtual worlds are rapidly becoming part of the educational technology landscape. Second Life (SL) is one of the best known of these environments. Although the potential of SL has been noted for health professions education, a search of the world’s literature and of the World Wide Web revealed a limited number of formal applications of SL for this purpose and minimal evaluation of educational outcomes. Similarly, the use of virtual worlds for continuing health professional development appears to be largely unreported.

**Methods:**

We designed and delivered a pilot postgraduate medical education program in the virtual world, Second Life. Our objectives were to: (1) explore the potential of a virtual world for delivering continuing medical education (CME) designed for physicians; (2) determine possible instructional designs using SL for CME; (3) understand the limitations of SL for CME; (4) understand the barriers, solutions, and costs associated with using SL, including required training; and (5) measure participant learning outcomes and feedback. We trained and enrolled 14 primary care physicians in an hour-long, highly interactive event in SL on the topic of type 2 diabetes. Participants completed surveys to measure change in confidence and performance on test cases to assess learning. The post survey also assessed participants’ attitudes toward the virtual learning environment.

**Results:**

Of the 14 participant physicians, 12 rated the course experience, 10 completed the pre and post confidence surveys, and 10 completed both the pre and post case studies. On a seven-point Likert scale (1, strongly disagree to 7, strongly agree), participants’ mean reported confidence increased from pre to post SL event with respect to: selecting insulin for patients with type 2 diabetes (pre = 4.9 to post = 6.5, *P*= .002); initiating insulin (pre = 5.0 to post = 6.2, *P*= .02); and adjusting insulin dosing (pre = 5.2 to post = 6.2, *P*= .02). On test cases, the percent of participants providing a correct insulin initiation plan increased from 60% (6 of 10) pre to 90% (9 of 10) post (*P*= .2), and the percent of participants providing correct initiation of mealtime insulin increased from 40% (4 of 10) pre to 80% (8 of 10) post (*P*= .09). All participants (12 of 12) agreed that this experience in SL was an effective method of medical education, that the virtual world approach to CME was superior to other methods of online CME, that they would enroll in another such event in SL, and that they would recommend that their colleagues participate in an SL CME course. Only 17% (2 of 12) disagreed with the statement that this potential Second Life method of CME is superior to face-to-face CME.

**Conclusions:**

The results of this pilot suggest that virtual worlds offer the potential of a new medical education pedagogy to enhance learning outcomes beyond that provided by more traditional online or face-to-face postgraduate professional development activities. Obvious potential exists for application of these methods at the medical school and residency levels as well.

## Introduction

### Background

Virtual worlds are rapidly becoming part of the educational technology landscape. Forterra’s OLIVE, The Croquet Consortium, Sun Microsystem’s Project Wonderland, ProtonMedia’s Protosphere, and Linden Lab’s Second Life are all examples of virtual world environments [1-5]. Platforms like these are potential environments for providing medical education.

Second Life (SL), one of the best known of these virtual worlds, consists of a flat-earth simulation of roughly 1.8 billion square meters, which would be about the size of Houston, Texas, if it were a physical place [6]. First launched in 2003, SL is an example of an immersive, three-dimensional environment that supports a high level of social networking and interaction with information. The SL virtual world is entered through a free client program called the SL viewer. Individuals enter SL as avatars that can take any form the user chooses. In the SL virtual world, residents can explore environments, meet and socialize with other residents (using voice and text chat), participate in individual and group activities, and learn from designed experiences. Built into the software is a three-dimensional modeling tool, based on simple geometric shapes that allows anyone to build virtual objects. These objects can be used, in combination with a scripting language, to add functionality [7].

While virtual worlds with their three-dimensional landscapes and customizable avatars seem similar to popular Massively Multiplayer Online Games (MMOGs), they do not adhere to the traditional definition of a game. Virtual worlds, like SL, are more focused on socializing, exploring, and building. As a result, there is an active educational community in SL. Over 300 colleges and universities have “builds” in SL where they teach courses and conduct research. A number of organizations, such as National Aeronautics and Space Administration (NASA), the National Oceanic and Atmospheric Association, National Institutes of Health, Jet Propulsion Laboratory, and National Public Radio, along with museums, educational groups, and a host of other government agencies, stage regular events, seminars, and workshops in Second Life [7].

Live sporting events, plays, meetings, seminars, research presentations, and musical concerts are all regular occurrences in Second Life. In 2008, Leong reported that the first virtual meeting of the International Virtual Association of Surgeons was held in Second Life, with 47 delegates attending from 5 countries [8]. There is no charge to create an SL account, although premium membership (which allows land ownership and greater technical support) is currently available for US$9.95/month. There is an economy in SL with the Linden dollar (L$) as the unit of exchange. Linden dollars can be used to buy, sell, rent or trade land, goods, or services.

Second Life demographics show that 83% of the population is 25 years or older, with the users over 44 years of age being the heaviest users on average. There is a close to even gender split among residents (57% male, 43% female), and more than 55% of the SL citizens come from outside the United States. In January 2008 residents spent a total of 28,274,505 hours in world and, on average, 38,000 residents were logged in at any particular moment. As of January 2009, just over 18 million accounts were registered (from over 150 countries), although there are no reliable figures for long-term, consistent usage [9,10,11,12,13].

Avatars are personalized by altering shapes, size, skin, hair, and clothing. They can be enlivened with animations to simulate facial expressions, posture, and gestures.

There are multiple channels for communication in the virtual world. Avatars can communicate by typing through local chat or instant messaging (IM), or by speaking through voice chat. Local chat is used for localized public conversation (called “local chat” or “backchat”) between two or more avatars and is visible to any avatar within a given distance. IMs are used for private conversation and do not depend on local proximity.

The virtual world is not constrained by real-world physics. This is an important consideration when constructing educational activities. Avatars can fly, float to observe goings-on from any angle, teleport in order to materialize in a different location, fall and recover, and change their visual perspective at will.

### Prior Work

Second Life has tremendous potential as a learning environment. The virtual world offers opportunities for student interaction, intense engagement, scripted immersive experiences, simulations, role-playing, and constructivist learning. The anonymity afforded by the avatar appears to lead to less inhibition and greater interaction. In addition, the greater sense of “presence” in a virtual world positively influences group process and cohesiveness, as well as engagement and attention [14,15,16]. We decided to use Second Life for our pilot since it is the most widely used virtual world platform and there is no charge to access it.

The problems with using a virtual world like SL for education and training lie mostly in the realm of technical and security issues. The software requires a download and has significant system requirements (processing power, up-to-date video card, and a fast broadband Internet connection) [17], the learning curve for navigation and interaction is steep, and the possibilities for technical problems and failures during the actual event are numerous. Many corporate or university firewalls do not allow access to public virtual worlds like Second Life.

Over the last three to four years, there has been growing interest in the medical and public health communities in using Second Life for public education, outreach, and training. There are a number of medical and health-related locations in Second Life. Most of these are education and awareness locations featuring kiosks and visual displays, health videos, slideshows, and Web links [18-26]. Beard (2009) reports 68 relevant health-related SL locations, 34 of which were designed to disseminate health information [27]. There are several medical simulation sites where nursing or medical students can practice with virtual equipment, procedures, or lab results [28-32]. For example, the Imperial College of London has created a game-based simulation in Second Life for undergraduate medical students where they can interact with virtual respiratory therapy patients in order to build their skills and confidence [33,34]. Reports that describe the potential of Second Life for health professions education are common [18,35], but a recent comprehensive review [27] identified only 11 actual programs. Our search of English language peer-reviewed publication databases did not identify any formal evaluation of the educational effectiveness of health professional training in SL or other virtual worlds. Recent reports have commented on this lack of empirical evidence of learning impact [20,35]. We did find evaluated virtual world experiences in other disciplines, such as an interdisciplinary communications course taught in Second Life [36]. We concur with that article’s author, Leslie Jarmon, that there are few empirical studies that inform instructional design and learning assessment in virtual worlds.

Physicians in the United States and in many other countries are required to undergo a specific number of hours of continuing medical education each year as a condition for maintenance of specialty board certification, and in some cases licensure, hospital admitting privileges, and insurance plan participation. The popularity and scope of available online, Web-based CME programs has increased dramatically in recent years [25,37,38], but has yet to include virtual worlds like SL as a venue.

### Purpose

The purpose of this project was to explore the potential of using a virtual world platform for medical education through the development of a one-hour, interactive seminar for postgraduate primary care physicians on the topic of insulin therapy for type 2 diabetes.

The objectives of this pilot study were to: (1) explore the potential of the virtual world, Second Life, for CME activities; (2) determine possible instructional design approaches for using SL for CME; (3) understand the limitations of SL for CME activities; (4) understand the barriers, solutions, and costs to using SL, including participant and presenter training; and (5) measure participant learning outcomes and feedback.

The learning objectives for the participating physicians were to: (1) learn to be aware of insulin inertia (in other words, not delaying initiation of insulin therapy when indicated); (2) learn to use glycemic patterns to choose starting insulin; (3) learn to decide among basal, prandial, and premixed insulins; and (4) gain confidence titrating insulin.

## Methods

### The Venue

We started with an existing Boston University School of Medicine Second Life build (or “sim”) [39] constructed on a private island owned and developed by Boston University School of Medicine (BUSM) for an earlier, joint project between BUSM and the World Health Organization (WHO). Ownership of the island allowed the developers to control access and thus provide security and privacy for the attending physicians. If an SL venue is not private, there is a risk of random avatars wandering into and potentially disrupting an event.

The existing BUSM/WHO meeting location was modified to add capacity, make it outdoors with no roof, open the walkways for easier navigation, and include automatic seating such that when avatars click on the seats, they automatically sit. We also built a media screen with a built-in script for controlling the PowerPoint slides that could also be used to project movies or websites.

### Recruitment

Since this was a pilot, the plan was to keep the attendance small. Institutional Review Board approval was obtained from BUSM. Participants were recruited from two family medicine listservs and from prior participants in our online CME courses. Given the anticipated time commitment, including training and pre and post surveys, participants were offered an honorarium for completion of all activities. Since this was a pilot and not an accredited CME program, no CME credits were provided. Participants were offered a Second life coaching session if they had no previous SL experience.

Initially, 41 physicians expressed interest in the program. Ongoing email contact helped to further qualify the candidates, arrange coaching sessions, clarify objectives, confirm hardware/software requirements, and send candidates to the online pre survey. Over the course of three weeks, the original 41 was pared down to 14. Some had conflicts with the workshop date, some did not have a compatible computer, and still others did not respond to emails.

Of the 14 participants, 8 were female and all were primary care physicians (family medicine specialty). Participants had spent an average of 16 years in practice and resided in seven different states (NC, IL, CA, MA, SC, CT, KY). One observer attended the session logged in from Geneva, Switzerland. Of the 14 participants, 4 reported having previously logged into Second Life, and 9 described themselves as “daily Internet users.”

The organizers commissioned Dr. Elliot Sternthal (MD, FACP, Director of Outpatient Diabetes Program, Boston Medical Center) to provide the session content. The instructional emphasis was to understand the options around diabetes patient assessment, insulin administration, and titrating dosage.

### Training

Of the 14 participating physicians, 3 were experienced Second Life residents who did not require coaching. The remaining 11, plus the seminar speaker, were coached in one-on-one sessions conducted by phone with coach and participant each on their respective computers. It required an average of 12 email communications, and an average of 78 minutes of coaching time per participant to gain required proficiency. A checklist of skills (see Textbox 1) to be mastered was the organizing framework for each coaching session.

**Key Second Life skills taught in training sessions**

**Getting started**

Logging on

Accessing the Second Life URL.

Do you have a computer gaming background?

Quit

**Orientation**

Camera controls: Understanding the various navigational commands allows residents to alter their viewpoint and focus in on specific items (eg, the slides in a presentation).

Mini map: This is a smaller map of the localized area in which a resident’s avatar is standing that helps find other nearby avatars or landmarks.

Big map: This is a map of the entire Second Life grid. Residents can use this to locate themselves, to search, to teleport to new locations, and generate URLS that guide others to specific locations.

Create landmark: Sets a physical “bookmark” so that residents can return to the same location.

Environmental settings: Used to control the time of day.

**Movement**

Teleport: Used to transport from one location to another.

Walk

Fly

Right click on an object: Used to obtain more information about an object or another avatar.

Sit

**Communicate**

Audio preferences

Text chat

IM (Instant Message) a specific person.

Talk

Right click to see profile.

Offer friendship: Used to establish a connection with another avatar so that residents can easily communicate with them and exchange goods or services.

Use of Skype/ headset use.

**Extras**

Take a snapshot: Takes a photograph of whatever is currently on the screen.

Inventory management: This is the place where residents’ virtual goods are stored (landmarks, clothing, objects, information cards). As the inventory grows, it is important to devise a filing system and a method for organizing it so that residents can easily find and retrieve desired items.

At the conclusion of the coaching session, participants were awarded a rating by one of the authors to indicate their degree of comfort with SL (1 = not comfortable, 2 = reasonably comfortable, 3 = very comfortable). The rating allowed us to partner confident doctors with less confident doctors. Partners were encouraged to arrange another meeting in SL with their partner prior to the workshop to practice navigational skills and camera controls. It was also hoped that knowing at least one other avatar would provide participants with a more comfortable experience at the actual event.

### Instructional Design

The organizers worked with Dr. Sternthal to create an instructional design for the hour-long session and build an avatar that resembled him in real life. Dr. Sternthal started with a PowerPoint deck to support a 40-minute insulin therapy talk. We found that this deck, which would work well for a face-to-face talk, was too long and did not allow opportunities for the interaction and activity afforded by the virtual world. The organizers worked with Dr. Sternthal to shorten the talk, focus in on the key concepts, add visual elements, increase the interactivity, and leverage the unique capabilities of Second Life. Strategic questions were inserted (answers to be given by the doctors in local chat) in order to surface misconceptions and points of confusion among the participants. We also decided to introduce two mock diabetes patients to the session in order to apply the session content to a real-world scenario. This entailed designing two age-appropriate, overweight avatars. During the actual session, Dr. Sternthal gave a lecture interspersed with active engagement junctures. At those points, Dr. Sternthal asked questions related to his lecture, interviewed the patients, displayed their lab results, and requested input from participant doctors on treatment plans.

**Figure 1:**
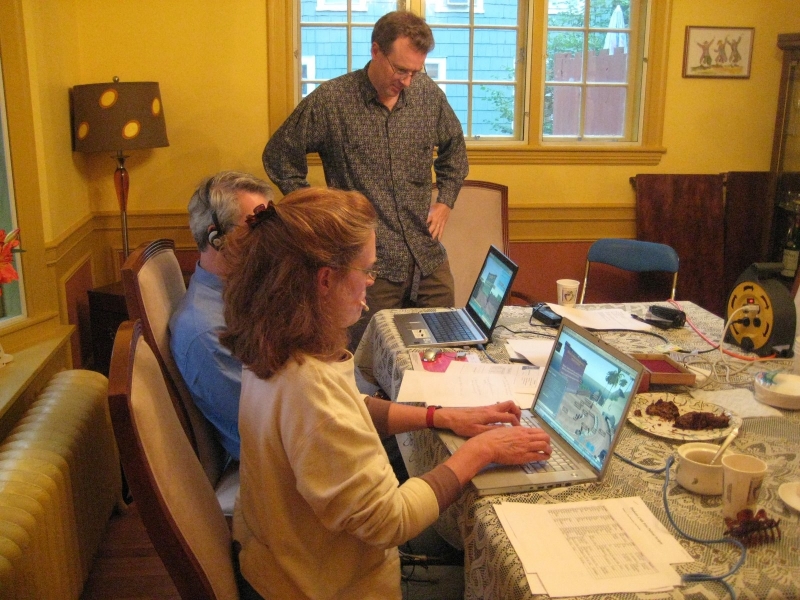
CME event team on the night of the event

Since the organizers and the speaker all lived in the same geographical area, we opted to conduct the seminar in the same physical location (see Figure 1) while the participating physicians were on their own computers in their homes or offices across the United States or in Switzerland. This allowed easy communication between the organizers during the event and convenient technical support for the speaker. As insurance against sound problems (one of the more often encountered SL technical issues), Skype [40] IDs were collected from all participants in advance so that a Skype conference call could be placed between the speaker’s location and any participants experiencing sound problems.

### The Backchat

One of the features of Second Life is the ability to conduct text-based local conversations that every avatar in the immediate vicinity can “hear” (read). For this pilot, we experimented with the use of backchat in a number of ways. First we used it as a vehicle to increase workshop interactivity. At predetermined points throughout the seminar, Dr. Sternthal asked for the doctor’s input (eg, “What would you recommend for this patient, given these lab values?”) We also used backchat as a way to solicit more information from the participants (eg, “How many diabetics do you see in your practice?” “What are the most often-cited patient reasons for not wanting to go on insulin?”) An unexpected advantage of the backchat that we noticed was that doctors talked to doctors throughout the event. Realizing the potential value of the backchat, we performed an analysis of its content. The seminar’s entire chat log (25 pages) was printed and then coded according to the following categories:

*Chatter: *greetings, compliments, farewells, and casual conversation not pertaining to the content (eg, “Hi! Good to see you.”)

*Technical: *questions or comments related to the functioning of Second Life (eg, “Remind me how to sit, please.”)

*Logistical: *questions or statements related to the logistics of the event (pre and post surveys, timing, location, etc) (eg, “Yes, I did complete the survey.”)

*Doctors answering questions: *statements given in answer to a question posed by the speaker (eg, “It depends on when the sugars are high.”)

*Doctors asking questions: *questions posed to the speaker (eg, “When we start insulin, how often do we follow-up?”)

*Doctors exchanging information: *instances of participants answering other participants’ questions, providing links, making suggestions to each other (eg, “Is there a difference in A1C between premixed insulin and NPH and regular?” Three other doctors answer, “Yes. Yes. Yes.”)

### Evaluation

Online surveys that included clinical skill confidence questions were completed before and following the session in order to assess physician reactions to the experience and evaluate learning transfer. These included two case studies before and two case studies after the session with questions designed to ascertain change in competence with respect to two learning objectives of the session: (1) how to properly initiate basal insulin in a patient with type 2 diabetes, and (2) how to initiate prandial (meal-time) insulin in such a patient. The cases were rated in a blinded fashion as either correct or incorrect by the two physician authors. This case approach has been shown to have good validity as compared to actual clinical practice [41].

### Statistical Analyses

The significance of the increase in the proportion of correct scores on the case studies was tested using the Fisher exact test due to the small cell sizes. Fisher exact tests were performed using Epi Info version 3.5.1 (Centers for Disease Control and Prevention, Atlanta, GA) [42]. Means of responses on the Likert scale items were tested with paired *t* tests. The change from pre to post in the distribution of the Likert scale responses to the confidence questions was tested using the Wilcoxon signed rank test with the Statistix statistical package (Analytical Software, Tallahassee, FL) [43].

## Results

### The Event

**Figure 2:**
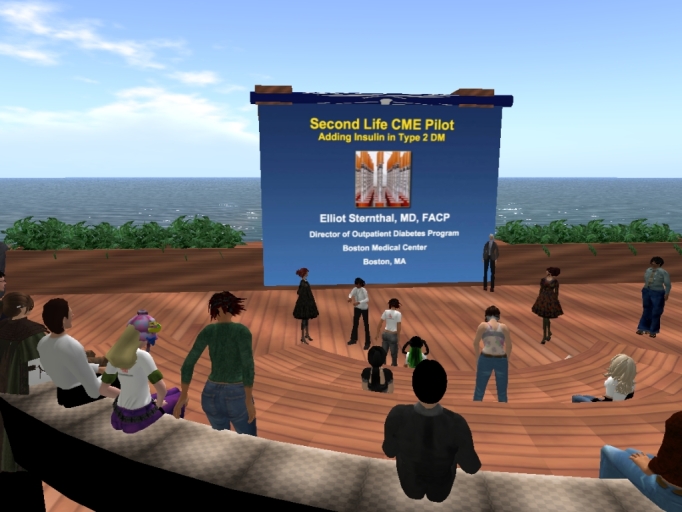
Avatars assembling just before the event on the Boston University/World Health Organization Second Life location

**Figure 3:**
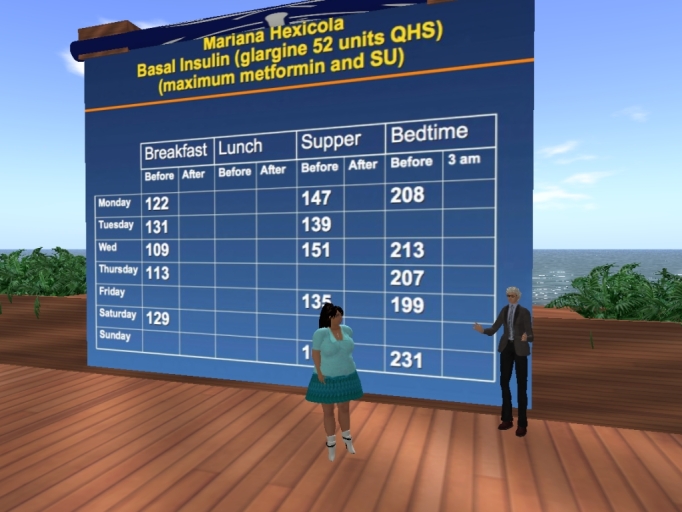
Dr. Elliot Sternthal, diabetes specialist, reviews lab results with fictional diabetic patient, Mariana Hexicola

The event was held on Monday, June 15, 2009, from 7:00pm to 8:00pm (EST). Avatars were asked to arrive 30 minutes early to get everyone settled and to resolve any technical issues. The session officially ended at 8:00pm, but the physicians stayed until 8:30pm asking questions and socializing. See Figures 2 and 3 for scenes from the actual event.

### Evaluation Outcomes

Of the 14 participant physicians, 12 rated the course experience, 10 completed the pre and post confidence surveys, and 10 completed both the pre and post case studies. 

The data showing pre-post changes in confidence regarding insulin therapy can be found in Table 1. After participation in this program, the pilot group reported a statistically significant increase in confidence in their ability to select, initiate, and adjust insulin for patients with type 2 diabetes.

On test cases, the percent of participants providing a correct insulin initiation plan increased from 60% (6 of 10) pre to 90% (9 of 10) post (*P*= .2), and the percent of participants providing correct initiation of mealtime insulin increased from 40% (4 of 10) pre to 80% (8 of 10) post (*P*= .09).

All participants agreed that this SL experience was superior to other online methods, and most also felt that the SL method was as good as, if not better than, face-to-face methods. All agreed they would take other CME events in SL. Textbox 2 displays representative comments from the physicians about their experience with the seminar. Textbox 3 displays representative participant comments explaining why they felt the course was superior to face-to-face CME courses. Textbox 4 shares insights from Dr. Sternthal regarding his experience with the CME event.

**Table 1:** Course evaluation: change in confidence and competence and rating of course quality

Statement / Answer Categories		Pre Course	Post Course	*P*^a^
**I am confident in my ability to select the appropriate insulin formulation and dose for starting a type 2 diabetic patient on insulin.**				
Mean (N=10)		4.9	6.5	.002
Median		5	6.5	
Strongly agree		1	5	.02
Agree		2	5	
Agree somewhat		5	0	
Neutral		0	0	
Disagree somewhat		1	0	
Disagree		1	0	
Strongly disagree		0	0	
**I am confident in my ability to initiate insulin therapy for my type 2 diabetes patients.**				
Mean (N=10)		5.0	6.2	.02
Median		5	6	
Strongly agree		1	2	.04
Agree		3	8	
Agree somewhat		4	0	
Neutral		0	0	
Disagree somewhat		1	0	
Disagree		1	0	
Strongly disagree		0	0	
**I am confident in my ability to appropriately adjust the insulin dose among patients with type 2 diabetes whom I have started on insulin.**				
Mean (N=10)		5.2	6.2	.004
Median		6	6	
Strongly agree		1	4	.04
Agree		5	4	
Agree somewhat		1	2	
Neutral		1	0	
Disagree somewhat		2	0	
Disagree		0	0	
Strongly disagree		0	0	


Evaluation of Second Life Experience as Potential CME^b^ Activity			Post Course	*P*^a^
	**Overall, I found this experience in Second Life to be an effective method of CME**			
	Strongly agree		6 (50%)	
	Agree		4 (33%)	
	Agree somewhat		2 (17%)	
	Neutral		0	
	Disagree somewhat		0	
	Disagree		0	
	Strongly disagree		0	
	**I would take other CME courses in Second Life.**			
	Strongly agree		9 (75%)	
	Agree		3 (25%)	
	Agree somewhat		0	
	Neutral		0	
	Disagree somewhat		0	
	Disagree		0	
	Strongly disagree		0	
	**The Second Life approach to CME was superior to other methods of online CME in which I have participated.**			
	Strongly agree		6 (50%)	
	Agree		5 (42%)	
	Agree somewhat		1 (8%)	
	Neutral		0	
	Disagree somewhat		0	
	Disagree		0	
	Strongly disagree		0	
	**This Second Life CME method is superior to face-to-face methods of CME.**			
	Strongly agree		2 (17%)	
	Agree		1 (8%)	
	Agree somewhat		3 (25%)	
	Neutral		4 (33%)	
	Disagree somewhat		2 (17%)	
	Disagree		0	
	Strongly disagree		0	
	**I would recommend to my colleagues to participate in a Second Life CME course.**			
	Strongly agree		6 (50%)	
	Agree		6 (50%)	
	Agree somewhat		0	
	Neutral		0	
	Disagree somewhat		0	
	Disagree		0	
	Strongly disagree		0	

^a^*P* value for change in means was determined with paired *t* test; *P* value for change in distribution of Likert scale responses was determined using Wilcoxon signed-rank test.

^b^This event was not a formally accredited continuing medical education (CME) activity.

**Qualitative feedback on the second life experience with medical education**

”**What are the strengths of this potential method of CME?**

“Invites interaction without intimidation…”

“No travel, sitting at home. No cost…”

“Interactivity…”

“Felt like a more active participant…”

“Fun!”

“The local chat – the input from the other members made it more like a discussion group…”

“You only have to connect into one program and it is all there. It feels like a group experience.”

**What were the most interesting elements of your experience?**

“Loved sitting at home while attending live…”

“Real-time, ongoing questions, clinical comments.”

“The discussions with the ‘patients’…”

“The ability to see my colleagues questions in real-time ...and have them answered.”

“The anonymity...”

“The local chat/running comments offered a great way to ask questions, while thinking of them, without interrupting the speaker…”

**What were the most confusing elements of your experience?**

“Some of the local chat was distracting…”

“No confusing elements. Everything was great and in easy format.”

“Getting onto the SL site…”

“Getting going took some time. Once I had my avatar and basic directions, it was pretty easy.”

“At first, it was difficult to concentrate on the lecture while people were typing questions and comments.”

*What are the weaknesses of this potential method of CME?*

“Perhaps hard to convince other new users that the interface is easy — it really was — but there is a barrier to overcome.”

“None that I can think of.”

“New technology, still a bit raw…”

“Navigating in SL takes practice.”

“If internet connection doesn’t work…”

“Need participants who are tech savvy and able to multitask.”

“Definitely a new type of ‘manners’ and courtesy that needs to be learned in this type of CME.”

“Steep learning curve for some…”

*What advice do you have for the developers?*

“None.”

“Have friends available for chat/email.”

“Have some very good health sites to give us…”

“Perhaps not use local chat?”

“Some sound issues (echo)…”

“It was all fine. Lots of help when needed…”

“If not for the ‘guide at the side’ I would not have been able to participate.”

“Add pauses…time for questions, discourage ‘chatting.’” “Could have been a bit abbreviated.”

*What did you learn about diabetes management that you didn’t know before?*

“Adding bolus insulin to largest meal first, then reevaluating need to add bolus to other meals.”

“New perspective on newer agents.”

“I feel more comfortable with formulations of insulin now.”

**Ways in which this course was deemed superior to face-to-face CME events: participants’ responses**

“Able to listen to more views on the topic from other participants in a very time effective manner (by the typed answers for the presenters questions).”

“I would say that the biggest advantage is being able to attend the conference from wherever and still feel as if you were actually present at the conference.”

“The presentation was very interactive. Instead of raising my hand and asking a question, I could type a question at any time and know that the presenter would eventually see it. I’m not the extroverted type, so don’t always ask a question when given the opportunity.”

“The ’patients’ made it interesting and helped apply what we had learned to common patient scenarios. It’s amazing how real it all feels during the presentation—a great way to learn.”

“I feel having an alter ego on SL facilitates learning because of the freedom of expression—less fear of being judged. Unlike face-to-face meetings, looks, mannerisms, and speech patterns are taken out of the experience for the learner. Instead, the learner relaxes in a pleasant environment (home) and can concentrate on learning.”

“This was one of the great experiences I have had. The phone or the other Web ones are a little boring. This one just kept me on my toes.”

“There was a nice element of play that enhanced the fun aspect of learning. I miss having fun in class.”

“Participants can communicate with each other during the session. No more saving questions till the end. The questions are addressed by all, not just the speaker, as they arise in the participants’ minds. This enhances learning.

**Comments from Dr. Sternthal: experience conducting the event**

This was indeed a unique experience for me. I have extensive experience in live face-to-face presentations to audiences of various sizes. I also have done numerous remote presentations via telephone and Web-based streaming where the audience is not visible just audible.

The SL presentation, while not face-to-face in the traditional sense, used a scenario that provided a sense of actually attending a meeting via the surrogate (avatar), thereby overcoming some of the sense of isolation (and often boredom) that is associated with just staring at remotely advanced slides on a monitor screen, typical of many remote programs. At the same time, with SL, there is a sense of anonymity and safety from critique and a loosening of inhibitions that facilitate the question and answer interaction. Therefore, SL seems to offer valuable features of both live face-to-face and remote programs: group interaction in a safe learning environment.

I would not consider moderating this SL program difficult. It was highly enjoyable and would not appear daunting to an experienced lecturer. I found certain challenges: coordinating the slides with the avatar patients, scanning and addressing in real-time the continuous chat questions and responses to clinical questions, and monitoring the overall flow of the program. It was important to keep things moving on time and to avoid prolonged silent times, so as to keep the participants engaged.

Interacting with the other avatars was not really different than fielding questions from a live audience. As I mentioned above, their presence gave a feeling of community and group dynamics to the presentation. The avatars all actively participated, likely because of the secure, non-judgmental learning environment.

The mock patients were invaluable and provided a concrete clinical correlate of the slide material. They reinforced the concepts being taught. In a way, they were like the live patients who used to grace medical lectures many years ago. However, since they are made up, there is no issue with confidentiality or privacy, and they can be constructed to portray the clinical situation that the lecture is addressing (in this program, deficiency in insulin initiation and titration).

I believe that learning was greatly facilitated by this SL virtual environment based upon the active participation, the number and quality of the questions, and the overall positive interactions with the presenter avatar and the other avatars.

The training that I received involved logistics in navigating to and through the SL virtual world, using and moving the avatar, and presenting information by speech, slides, and keyboard. This was accomplished in 3 sessions followed by a dry run of the whole presentation.

I believe that SL virtual learning is effective, innovative, stimulating, and fun. Its format encourages active reflection and participation, and the mock patients provide the opportunity to try out various clinical maneuvers, increasing the chance that the desirable behavioral change will be adopted by the health care participant. This has been a chronic problem with CME programs: effecting the appropriate behavioral change. It will be very valuable to evaluate the incorporation and durability of these take-home messages in participants after attending an SL medical education program.

### Backchat Outcomes

In reviewing the backchat script, we recorded numerous incidences of doctors posing questions that were then answered by other participants, thus providing additional information over and above the seminar’s planned content. One participant noted, “The input from the other members made it more like a discussion group…”

The results regarding the backchat are shown in Table 2, and examples of typical doctor-to-doctor exchanges are shown in Textbox 5. A significant amount of backchat was devoted to doctors answering questions from the speaker. This reflects the seminar’s instructional design. In addition to this confirming information, there are a number of interesting trends revealed in the backchat. Almost all of the “chatter” (exchanges like ”Hello, how are you?” “Thank you, it was great!”) occurred at the beginning and end of the session. Almost all of the technical talk (eg, “How do I sit down?” “I can't hear.”) happened in the first 30 minutes before the session started. The doctor-to-doctor exchanges were quite robust. For example, doctors provided insight from their own clinical practice, talked about specific patient examples, and pasted URLs into the backchat to help other doctors find further information online.

**Table 2:** Backchat communication during educational session

Topic of text communication during educational session	Number of lines posted by topic	% of total lines posted
Technical	221	32
Chatter	197	29
Doctors answering speaker	132	19
Logistics	65	9
Doctors asking speaker	52	8
Doctors exchanging	19	3
Total	686	100

**Representative backchat excerpt**

Is the patient eligible for any assistance? Medicaid?

Maybe contact the drug companies; they have freebies for poor patients.

Could try premix NPH/regular.

Premixed 70/30 big, generic, cheaper than Lispro/Glargine combo.

Are there less expensive insulin formulas?

I agree with Abigail.

SU and metformin are $4/mon generic at certain pharmacies.

Any employer based options? A wellness program? Can we get the number of blood sticks down?

But she needs insulin.

While the backchat was used to increase the session interactivity, not all of the participants found it helpful. A few participants noted that it was distracting at times, and one suggested limiting the amount in future actual CME sessions. One possible explanation for the feeling of distraction is that, in SL, the backchat appears momentarily on top of the action on the screen. In future CME events, we should prepare the participants for this and provide advice for managing the information flow.

### Developer Investment

Since one of the pilot objectives was to understand the time required to design and build a virtual world experience, careful record was kept of the organizers’ hours. This is summarized in Table 3.

**Table 3:** Time spent developing and running the CME event

Item	Hours required for activity
**Developer activities**	
Planning	15
Instructional design	12
Coaching doctor participants	20
Venue build (work with builder, troubleshooting, creating signage)	10
Avatar design	10
Communications (email, telephone, meetings)	16
Technical issues/security	8
Rehearsals	6
Event	3
Project director effort	8
**Speaker activities**	
Rehearsals	6
Event	3
**Total person-hours**	109

## Discussion

### Principal Results

Overall, this pilot was very successful. The participant physicians’ responses indicated that this was a positive and engaging experience that could meet their CME needs and fit their busy schedules. All respondents agreed it was superior to other methods of actual online CME they had experienced. In comparison with face-to-face CME, one-half of the respondents agreed Second Life CME was superior, one-third were neutral, and only two respondents disagreed “somewhat.” This is a remarkable endorsement of such a new educational method. Our results showed that the virtual world model can have a positive impact on learner self-efficacy and, based on gains demonstrated from the cases, suggested a potential positive impact on clinical competence as well.

Many of the participants noted the convenience of an online seminar as one of the most important advantages—no travel is required, and they can participate from the comfort of their own homes. Since other online methods of instruction also offer this convenience, what justifies the expense of a virtual world course?

This pilot points to at least two important virtual world advantages: (1) the added sense of presence afforded by a representative avatar, and (2) the added real-life application provided by mock-patients [14,44]. Judging by the participant comments, the injected realism of mock patients was effective and added to the seminar’s impact. These methods required considerable dexterity on the part of the speaker, Dr. Sternthal. The speaker must maintain fidelity to the planned script but, at the same time, pay attention to the backchat, adjust comments to answer participant questions, role play with the mock patient, and roll with whatever technical limitations, unexpected results, or problems arise. It will be worthwhile to explore and develop future instructional design options using mock patients.

### Coaching Session Lessons

Relevant information emerged from the pre-event Second Life coaching sessions. There was tremendous variability in the participants’ Second Life learning curve, with some quickly mastering the navigation and trying out functionality on their own, while others were very uncertain and hesitant to try buttons or commands. In hindsight, not all items on the skills checklist were needed for the event. Since the Second Life learning curve is so steep, it might be more effective to narrow the training to those skills absolutely required for the event and trust that the participant will go further with the virtual world on their own time, if they are interested.

Reasons for the SL orientation problems experienced by the doctors can be grouped into two categories, difficulty understanding the metaphor and technical skills:

*1. Difficulty understanding the metaphor.* Many doctors had trouble understanding where they were when training in the virtual environment. This was expressed by questions like, “Who is that?” (meaning the trainer’s avatar), “What is it we’re trying to do here?” “I’m confused about where I am,” and repeated instances of participants going to the Second Life website [5] instead of opening the Second Life application in order to enter the virtual world.

*2. Technical skills.* For some doctors, mastering the menus, keyboard commands, and interface elements was no problem at all. Others had significant trouble remembering how to perform basic tasks such as sitting, chatting, accessing inventory, and setting landmarks. The variability in coaching time reflects these differences and is most likely related to the time each participant has spent online and/or working with other applications. It was noted that physicians with gaming experience were much more adept at mastering the basics.

It was also interesting to note the differences in approach to learning. Some of the physicians needed to understand the context for any navigational command (“Why would you need to take a snapshot in Second Life?” or “When would you use that function?”), while others were perfectly content to run through a list of skills and tick them off with no need to attach relevance.

We observed that subjects tended to choose avatars that looked like them in real life. For example, one African American participant explained, “But I need the avatar’s skin to be darker.…” This is consistent with previous studies [44,45] showing that people choose avatars for self-representation, based primarily on how similar avatars are to themselves. It also implies the increased sense of presence afforded by the virtual world [14].

The tactic of partnering confident with less confident subjects for additional practice met with modest success but could be used more effectively in the future if there was more time between the coaching session and the event.

### Overall Lessons Learned

Since this pilot was primarily designed to develop expertise in designing and running CME and other medical education events programs in the virtual world, it is important to capture key lessons for future programs.

Among the lessons learned is that an event like this has to be designed in such a way that it answers the question “Why SL?” before it gets asked. In other words, it is critical to take advantage of the unique opportunities afforded by the virtual world to push the experience beyond what could be delivered via a website or a webinar.

With so many opportunities to ”go wrong” with a technically complex platform, it is clear that thorough planning is paramount. This event was meticulously planned, rehearsed, and buttressed with contingency plans.

The time investment to plan, prepare, and deliver a one-hour event is steep, and the learning curve for the doctor-participants is not trivial. From our experience, delivering a CME event in Second Life is much more time consuming than face-to-face or webinar events.

Virtual world events require skilled and unflappable speakers. Speakers must be knowledgeable, confident in their expertise, unruffled with the inevitable technical glitches, good humored, in tune with the students, and able to field questions from the backchat while still keeping an eye on content and timing.

Overall, there is a certain excitement that comes from just being there, all together, in the virtual world. The physician participants are more forthcoming, brave, involved, and present. There is an interesting ”protection“ effect that comes with the avatar anonymity that promotes disclosure and sharing. Many of the doctors commented on it, and from the backchat logs, one could certainly sense an openness and willingness to venture further. In fact, the informal exchange and learning resulting from the backchat suggests a less top-down and more Socratic approach to learning and instruction with participants and experts exchanging strategies and ideas more fluidly. What’s more, the backchat exchange might result in enhanced professional connections among participants that could extend beyond the boundaries of the single event.

But while the backchat offers an opportunity for increased interactivity, it can be distracting. Organizers should prepare participants for this and set ground rules about keeping the chat to a professional level.

Lastly, it is important to leverage the more playful aspects of Second Life without drifting into unprofessional behavior. In this event, the mock-patient avatars’ responses, the 15-minute conversational warm-up before the session started, and a social phase (including “serving” champagne) at the end all helped to serve that purpose.

### Comparison with Prior Work

Prior reports have described the potential of SL and other virtual worlds for the training of health professionals, or have described a curriculum or program in SL [24,33,34,46,47,48]. Most of these reports, however, have not included feedback from the health professionals who were involved in this training. One report described improvement in leadership skills after participation in a trauma exercise in a non-Web-based virtual environment created by the investigators for a local client [32].

However, we did not locate prior peer-reviewed publications that described the impact of training delivered in virtual worlds like SL on health professionals’ confidence or on health professionals’ knowledge and competence in clinical management.

### Limitations

Chief among concerns for future CME programming is the time required to bring all participants to a functional level and the technical requirements/potential problems with the software. The time investment to scale the course must be considered. One hour per participant in one-on-one coaching sessions is not scaleable to larger enrollments. Future events will require group or self-directed training, and the training should be limited to the skills required for the event. While the technical requirements to run a virtual world are steep, it is clear that ever-increasing processing power and bandwidth will make this less of an issue going forward. Security concerns around virtual worlds will most likely be addressed with the development of platforms for installation behind firewalls and integration with other native systems. The potential for technical failure in this particular virtual world platform is high. System crashes and sound problems are the most commonly experienced issues. It is essential to have back-up plans for each possible technical snafu.

Although the improvements in performance on the case scores were large, our small sample size limited our ability to assess the statistical significance of these score changes. More robust evaluation work with larger samples is needed to measure impact on clinical skills and outcomes and to compare the relative effectiveness and efficiency of educational methods. Such work also needs to address the rapidly developing diversity of online approaches including virtual worlds. Also, further careful evaluation work is needed on less selected populations to determine if the enthusiastic reception of this SL method is replicable more broadly.

### Conclusions

The organizers are very optimistic about future use of virtual worlds applied to all phases of medical education including continuing medical education. The enriched environment, convenience, and possibilities for constructivist approaches all add up to tremendous potential. What’s more, it is expected that virtual worlds will become more ubiquitous in other types of computing. Some speculate that virtual worlds will soon replace our Internet browsers [30].

There are many instructional design possibilities to consider that will further leverage the unique advantages of these innovative technological environments [49]. In future events, we will consider expanding the mock patient element so that physician pairs (or teams) could interview their own mock patients and compare findings. This should enhance learning on the psychosocial aspects of patient care, allowing doctors to interact with other avatars (including patients, staff, and experts) in a safe, simulated environment and reflect on their learning. The backchat could be used more specifically to boost engagement and information transfer. Simulated physiology models could be created to provide a deeper understanding of the biology behind the disease state. We intend to further investigate the unique potential of Second Life as a learning environment.

The results of this pilot suggest that virtual worlds offer tremendous opportunity to provide a space for constructivist learning at its best and to enhance learning outcomes beyond that provided by traditionally designed CME courses.
